# Polyploid giant cancer cells with budding and the expression of cyclin E, S-phase kinase-associated protein 2, stathmin associated with the grading and metastasis in serous ovarian tumor

**DOI:** 10.1186/1471-2407-14-576

**Published:** 2014-08-08

**Authors:** Hongcheng Lv, Yang Shi, Li Zhang, Dan Zhang, Guang Liu, Zhengduo Yang, Yan Li, Fei Fei, Shiwu Zhang

**Affiliations:** Department of Pathology, Tianjin Union Medicine Center, Tianjin, 300121 P.R China; Department of Colorectal surgery, Tianjin Union Medicine Center, Tianjin, 300121 P.R China; Department of Gynaecology and Obstetrics, Tianjin Union Medicine Center, Tianjin, 300121 P.R China

## Abstract

**Background:**

We previously reported that polyploid giant cancer cells (PGCCs) exhibit cancer stem cell properties and express cell cycle-related proteins. HEY PGCCs induced by cobalt chloride generated daughter cells and the daughter cells had a strong migratory and invasive ability. This study is to compare the expression of cyclin E, S-phase kinase-associated protein 2 (SKP2), and stathmin between PGCCs with budding and control HEY cells, and determine the clinicopathological significance of cell cycle-related protein expression in ovarian tumors.

**Methods:**

We used western blot and immunocytochemical staining to compare the expression levels of cyclin E, SKP2 and stathmin between PGCC with budding daughter cells and control HEY cells. In addition, immunohistochemical staining for cyclin E, SKP2 and stathmin was performed on a total of 80 paraffin-embedded serous ovarian tumor tissue samples. The samples included 21 cases of primary high-grade carcinoma (group I) and their metastatic tumors (group II), 26 cases of primary low-grade carcinoma without metastasis (group III), and 12 cases of serous borderline cystadenoma (group IV).

**Results:**

Single PGCC with budding in the stroma showed high correlation with the metastasis of ovarian carcinoma. Group I had a significantly higher number of single PGCCs with budding in the stroma than group III (85.71% [18/21] vs. 23.08% [6/26] cases; *χ*^2^ = 18.240, *P =* 0.000). The expression of cyclin E, SKP2, and stathmin was compared among the four groups. The expression levels of cyclin E, SKP2, and stathmin increased with the malignant grade of ovarian tumors and group II had the highest expression levels. The expression of cyclin E (*χ*^2^ = 17.985, *P =* 0.000), SKP2 (*χ*^2^ = 12.384, *P =* 0.000), and stathmin (*χ*^2^ = 20.226, *P =* 0.000) was significantly different among the 4 groups.

**Conclusions:**

These data suggest that the cell cycle-related proteins cyclin E, SKP2, and stathmin may be valuable biomarkers to evaluate the metastasis in patients with ovarian serous carcinoma.

**Electronic supplementary material:**

The online version of this article (doi:10.1186/1471-2407-14-576) contains supplementary material, which is available to authorized users.

## Background

Ovarian cancer (OC) is the fourth leading cause of cancer-related death among women in the United States. Ovarian serous carcinoma (OSC), the main histologic type of epithelial OC, has a poor 5-year overall survival rate [1]. Understanding the molecular mechanisms of ovarian carcinogenesis and metastasis is critical for the clinical diagnosis, treatment and prognosis evaluation [2]. Although, in most cases, the exact causes of OSC are unknown, the risk of developing OSC appears to be affected by several factors including familial and genetic factors, hormonal alterations, number of births, work-related stress, and environmental pollution [3–6]. Surgical excision and chemotherapy are the main treatment options for OSC. Chemoprevention holds promise for reducing cancer incidence and overcoming problems associated with the treatment of late-stage cancers [7]. However, OSC is associated with relatively high mortality rates because it lacks clear early detection or screening test, which means that many cases are diagnosed at advanced stages [8].

Polyploid giant cancer cells (PGCCs) are a special subpopulation of cancer cells that contribute to solid tumor heterogeneity and show significant variation in nuclei shape and number. We have previously demonstrated that PGCCs induced with cobalt chloride (CoCl_2_) exhibit cancer stem cell properties and asymmetrically generate daughter cells via budding. By using iTRAQ proteomic analysis and immunohistochemical staining, we found that HEY PGCCs with budding daughter cells abnormally express cell cycle-related proteins compared with diploid HEY cancer cells. Expression levels of cyclin E and cyclin D1 were markedly higher in purified HEY PGCCs than those in the control HEY cells. PGCCs with budding showed the highest expression of cyclin-dependent kinase (CDK) 2 and cyclin B1 [9]. Furthermore, the daughter cells derived from PGCCs showed a stronger migratory and invasive ability than untreated diploid cells. Animal experiments also confirmed that tumors derived from PGCCs had a higher nucleus-to-cytoplasm ratio and displayed mesenchymal changes compared with tumors derived from control HEY cells [10]. Based on iTRAQ proteomics analysis, western blot and immune staining, we confirmed that the expression of Cyclin E, SKP2, Stathmin in HEY PGCCs with budding daughter cells were higher than those in control HEY cells, which may provided new insight into how PGCCs and regular cancer cells are coordinately regulated in the progression of human ovarian carcinomas.

The cell-cycle related protein family consists of cyclins, CDKs, and cyclin-dependent kinase inhibitors (CDKIs). Cell cycle-related proteins play important roles in carcinogenesis, tumor development, and metastasis. Cyclin E forms a complex with CDK2 to regulate the progression of the cell cycle from the G1 to the S phase. This is the initial step in DNA replication and cell proliferation. Exogenous stimulators or abnormal molecular signals lead to upregulation of cyclin E expression, which shortens the G1 phase and allows the immediate entry of cells into the S phase. This alteration in the cell cycle increases cell proliferation and subsequent tumor formation. Lee et al. evaluated cyclin E expression in 78 cases of OSC, 72 cases of ovarian cystadenoma, and 55 cases of benign ovarian tumors [11]. They found that highest cyclin E protein expression was in OSC, followed by ovarian cystadenomas and benign ovarian tumors. These results suggest that the expression of cyclin E is positively associated with the development and histological grade of OSC. Davidson et al. reported that the cyclin E protein was overexpressed in OSC and associated with poor prognosis [12]. Together, these studies indicate that cyclin E may be a useful prognostic indicator for OC. Stathmin is involved in microtubule depolymerization. It promotes microtubules depolymerization or prevents microtubule polymerization in a phosphorylation-dependent manner during different stages of the cell cycle. Stathmin plays an important role in carcinogenesis, and it is highly expressed in breast cancer [13], prostate cancer [14], endocrine tumors [15], and ovarian carcinoma [16]. The expression of stathmin is closely related with cancer development and patient prognosis. S-phase kinase-associated protein 2 (SKP2) is a member of the F-box protein family, which specially recognizes and binds to phosphorylated substrates such as P27, P21, and E2F. SKP2 regulates the cell cycle mainly through the ubiquitin-proteasome pathway [17]. The expression of SKP2 has been closely associated with cancer development and metastasis [18]. Chiappetta et al. demonstrated that SKP2 overexpression was positively associated with the development of thyroid carcinoma [19]. Hung et al. reported that SKP2 protein overexpression increased cancer invasion and metastasis [20].

Many studies have described the expression of cyclin E, SKP2, and stathmin in OCs and investigated the correlation between cyclin E, SKP2, and stathmin expression and the clinicopathological characteristics of OC. Cell cycle-related proteins have been shown to induce PGCC formation and generate daughter cells with strong migratory ability. This study compared the expression of cyclin E, SKP2, and stathmin between PGCCs with budding and control HEY cells. We also determined the clinicopathological significance of cell cycle-related protein expression in OC.

## Methods

### Cancer cell line and culture

The human OC cell line HEY was purchased from the American Type Culture Collection (USA) and maintained in complete Eagle’s minimum essential medium (EMEM) supplemented with fetal bovine serum and antibiotics (100 U/mL penicillin, and 100 μg/mL streptomycin).

### Generation of PGCCs

HEY cells were cultured in complete EMEM in T75 flasks until they reached 90% confluence. Cells were treated with 450 μM of CoCl_2_ Sigma-Aldrich, St. Louis, MO, USA) for 48 h, as described previously [10]. After rinsing with 1× phosphate-buffered saline (PBS), the cells were cultured in regular EMEM. Most regular-sized HEY cells died following CoCl_2_ treatment, whereas scattered PGCCs survived the CoCl_2_ treatment. Ten to 14 days later, PGCCs (1 × 10^4^) with newly budding daughter cells (1 × 10^5^) were used for western blot analysis and immunocytochemical staining.

### Western blot analysis

Western blot analyses were done as described previously [9]. Cell extracts obtained from CoCl_2_-treated control HEY cells, HEY PGCCs (10%), and HEY PGCCs with budding cells (90%) were lysed in ice-cold buffer. The proteins were separated on a 10% sodium dodecyl sulfate-polyacrylamide gel and transferred to a polyvinylidene fluoride membrane (PVDF Membrane; GE Healthcare, USA). The membranes were blocked with 5% nonfat milk in 1× tris-buffered saline with 0.1% Tween-20 for 1 h at room temperature, incubated with mouse anti-cyclin E (1:500 dilution; SC-247, Santa Cruz Biotechnology) and rabbit anti-SKP2 (1:100 dilution; SC-7164, Santa Cruz Biotechnology) antibodies overnight at 4°C, and then with the appropriate secondary antibody for 1 h at room temperature. Protein expression was detected by using mixed ECL Plus reagents (RPN2132OL/AK, GE Life Sciences Co.) and the X-OMAT 2000 film processor. β-actin was used as a protein loading control.

### Tissue samples

Paraffin-embedded human OSC tissue samples accumulated between 2005 and 2013 were obtained from the Tumor Tissue Bank of the Tianjin Union Medicine Center. None of the patients had been treated before surgical excision. OSCs were graded according to the two-tier system, which is based primarily on the assessment of nuclear atypia, with the mitotic rate used as a secondary feature [21] and the information of TNM staging system for these OSC listed in Additional file 1: Table S1. The tumor diagnosis was verified by two pathologists. Cases of high-grade OSCs with metastasis, low-grade OSCs without metastasis, and serous cystadenomas were included in the study. The tumors were divided into 4 groups according to their pathologic characteristics: groups I and II, 21 cases of primary cancer (patient mean age of 57.57 ± 10.59, mean tumor size 149.21 ± 221.05 mm^3^) and their corresponding metastatic tumors (mean tumor size, 127.55 ± 221.25 mm^3^); group III, 26 cases of primary cancer without metastasis (patient mean age of 56.77 ± 10.80, mean tumor size, 624.22 ± 772.49 mm^3^); and group IV, 12 cases of borderline serous cystadenomas (patient mean age of 44.75 ± 18.19, mean tumor size, 769.69 ± 1502.98 mm^3^). The study was approved by the Tianjin Union Medicine Center Research Committee, and the confidentiality of patient information has been maintained.

### Tissue microarray

Formalin-fixed, paraffin-embedded tissues from the OC samples were stained with standard hematoxylin and eosin, and tumor tissues without necrosis were used to construct a tissue microarray with 1.5 mm cores (2.0 mm between cores). Two cores from every tumor sample were included in the tissue microarray. The tissue microarray block was sectioned for immunohistochemical (IHC) staining.

### Immunocytochemical (ICC) and IHC staining

ICC and IHC staining was performed using an avidin-biotin-peroxidase complex as described previously [22]. For ICC staining, HEY PGCCs with budding and control HEY cells were grown on glass coverslips until 70% confluence, washed with PBS, and fixed with cold 75% ethanol for 10 min on ice. The cells were incubated in 0.3% hydrogen peroxide for 10 min and then in 1.5% normal goat serum to block endogenous peroxidase activity and nonspecific protein binding. The cells were incubated with rabbit monoclonal anti-stathmin antibody (1:100 dilution; Epitomics, USA) overnight at 4°C in a humidified chamber. The following morning, the cells were incubated with biotinylated goat anti-mouse IgG for 30 min and counterstained with hematoxylin. For IHC staining, 4-μm-thick sections were deparaffinized in xylene and incubated in 3% hydrogen peroxide to block endogenous peroxidase activity. Sections were washed with PBS and heated in citrate buffer (0.01 M of citric acid, pH 6.0) for 20 min at 95°C in an autoclave. After blocking nonspecific binding sites with 10% normal goat serum, sections were incubated overnight at 4°C with mouse monoclonal anti-cyclin E (1:50 dilution; MAB-0019, Maixin. Bio, Fujian, China), mouse monoclonal anti-SKP2 (1:50 dilution, ZM-0454, Zhongshan Inc., Beijing, China), and rabbit polyclonal anti-stathmin (1:50 dilution; RMA-0641, Maixin.Bio, Fujian, China,) antibodies. Following incubation, the sections were rinsed with PBS, incubated with biotinylated IgG for 20 min at 37°C, incubated with 3, 30-diaminobenzidine chromogen for 1–3 min, and then washed with distilled water. Finally, all sections were counterstained with hematoxylin, dehydrated, and mounted.

### ICC and IHC scoring and quantification

The evaluation of cyclin E, SKP2, and stathmin expression was quantified according to the method described by Sun et al. [23]. Both the intensity and percentage of positive cells were evaluated. Staining intensity was scored as follows: 0, no staining; 1, weak positive (faint yellow staining); and 2, strong positive (brown staining). The number of positive cells was visually evaluated and stratified as follows: 0 (negative), <10% positive cells; 1 (weak), <30% positive cells; 2 (moderate), <50% positive cells; and 3 (strong), >70% positive cells. The sum of the staining intensity and positive cell scores was used to determine the staining index for each section.

### Statistical analysis

SPSS 13.0 statistical software was used for all statistical analyses. A two-sided *P-*value of <0.05 was considered significant. The chi-squared test was used to compare differences in cell cycle-related protein expression between the groups. The Wilcoxon rank test was used to compare the correlation between the expressions of different protein in two different groups.

## Results

### CoCl_2_-induced PGCC formation

**Figure 1:**
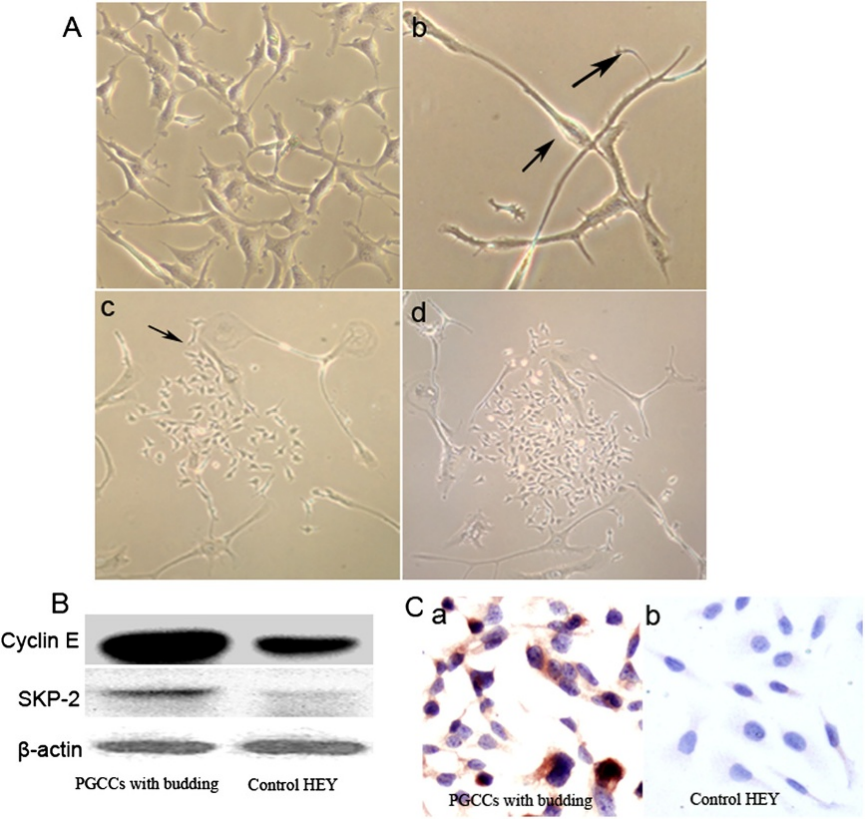
**PGCCs with budding daughter cells. A**. HEY PGCCs and control HEY cells. **a**. Control HEY cells (×400). **b**. HEY PGCCs induced by treatment with 450 μM of CoCl_2_ for 48 h (small black arrow heads PGCCs; large black arrow heads budded daughter cells from PGCC; ×400). **c**. PGCCs generated daughter cells via budding (black arrow heads budded daughter cells from PGCC; ×100). **d**. PGCCs use budding for renewal and fast reproduction. Cells in panel 1**A-c** were cultured in complete medium for 8 h (×100). **B**. Western blot of cyclin E and SKP2 expression in HEY PGCCs with budding and control HEY cells. **C**. ICC staining of stathmin in HEY PGCCs with budding and control HEY cells (×200).

We previously confirmed that diploid cells were selectively killed by high concentrations of CoCl_2_, whereas PGCCs survived from CoCl_2_ treatment. Compared with the HEY cells without treatment (Figure 1A-a), treatment of HEY cells with a high CoCl_2_ concentration (450 μM) for 48 h killed most diploid cells, whereas PGCCs could be clearly visualized after removal of floating dead cells. PGCCs were obviously larger than control HEY cells (Figure 1A-b). Surviving PGCCs cultured in media with 10% serum generated daughter cells 10–14 days after CoCl_2_ treatment. Figure 1A-c shows that 60% of the cells were regular-sized cells and 40% were PGCCs. PGCCs generated daughter cells via budding. The number of regular-sized cells dramatically increased from 60% to 90% after 8 h of continuous culture in complete medium, whereas the number of PGCCs decreased from 40% to 10% (Figure 1A-d). These cells were analyzed for cell cycle-related protein expression.

### Cell cycle-related protein expression in control HEY cells and budding PGCCs

Total proteins were extracted from control HEY cells and HEY PGCCs with budding. Western blot analysis showed that the expression levels of cyclin E and SKP2 were higher in HEY PGCCs with budding than in control HEY cells (Figure 1B). PGCCs with budding cells were trypsinized and grown on coverslips for 24 h, and then fixed with 75% ethanol for ICC staining. The expression of stathmin was higher in PGCCs with budding (Figure 1C-a) than in control HEY cells (Figure 1C-b).

### Clinicopathological significance of single stromal PGCCs in human OSC

**Figure 2:**
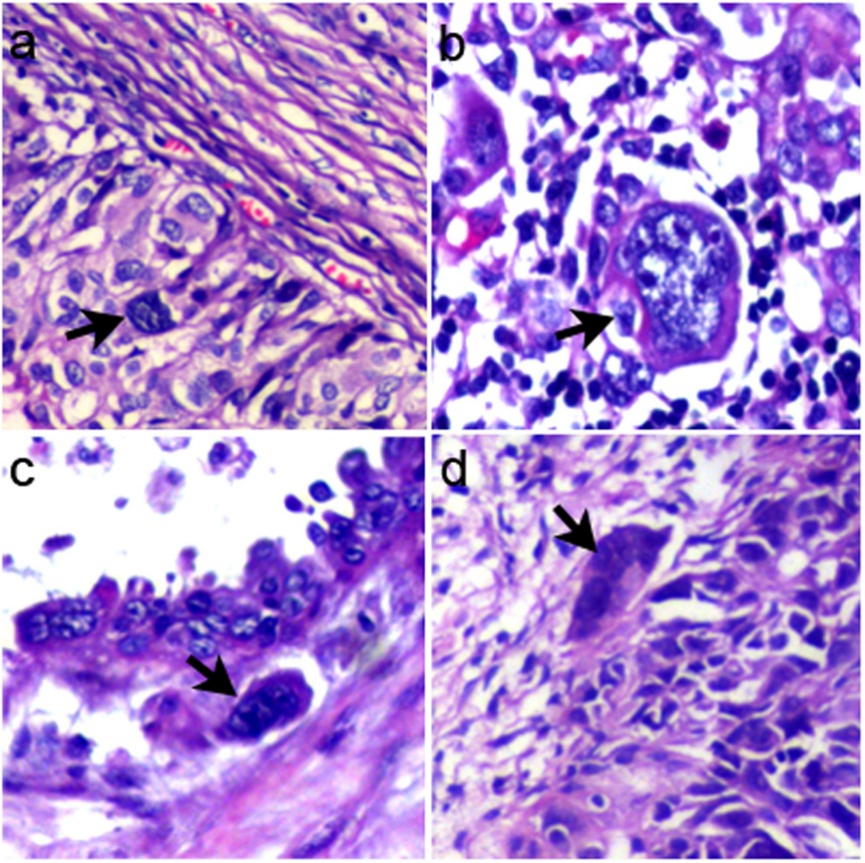
**PGCCs in OSC. a**. PGCCs in low-grade OSC (×200). **b**. PGCCs in high-grade OSC (×200). **c**. Single PGCC located in the invasive front of low-grade OSC (×200). **d**. Single PGCC located in the stroma of high-grade OSC (×200).

By using the definition of PGCCs set by Zhang et al. that characterized a PGCC as a cancer cell with a nucleus of at least three times larger than that of a diploid cancer cell [10], it was observed that PGCCs with giant or multiple nuclei were present in both low-grade (Figure 2a) and high-grade human OSCs (Figure 2b). The shape of PGCC nuclei was irregular. In OC tissues and metastatic tumors, the size of the PGCC nuclei was 10–20 times larger than that of regular diploid cancer cell nuclei (Figure 2b). Interestingly, single PGCC invaded into the stroma. Figure 2c and d show a single PGCC invading into the stroma in low-grade and high-grade OSCs, respectively. Single PGCCs invading into the stroma were highly associated with tumor metastasis (Table 1). Single PGCCs invading into the stroma appeared in 18 of 21 high-grade OSCs and 6 of 26 low-grade OSCs. This difference in the number of single PGCCs in the stroma between low-grade and high-grade OSCs was statistically significant (*χ*^2^ = 18.240, *P* = 0.000) (Table 2).

**Table 1:** **Profile of single stromal PGCCs and lymph node metastasis in ovarian tumors**

			Lymph node metastasis	
			Yes	No
Primary ovarian tumor with metastasis	Single stromal PGCCs	Yes	18	0
		No	3	0
Primary ovarian tumor without metastasis	Single stromal PGCC	Yes	0	6
		No	0	20
Borderline serous cystadenoma	Single stromal PGCC	Yes	0	0
		No	0	12

**Table 2:** **The differences of the percentage of tumor with single PGCC in the stroma**

	Group	n	The percentage of tumor with single PGCC in the stroma	***χ*** ^2^	***P***
Primary ovarian tumor with metastasis	I	21	85.71% (18/21)	18.240	0.000
Primary ovarian tumor without metastasis	III	26	23.08% (6/26)		

### Expression of SKP2, cyclin E, and stathmin was associated with OSC grade

**Figure 3:**
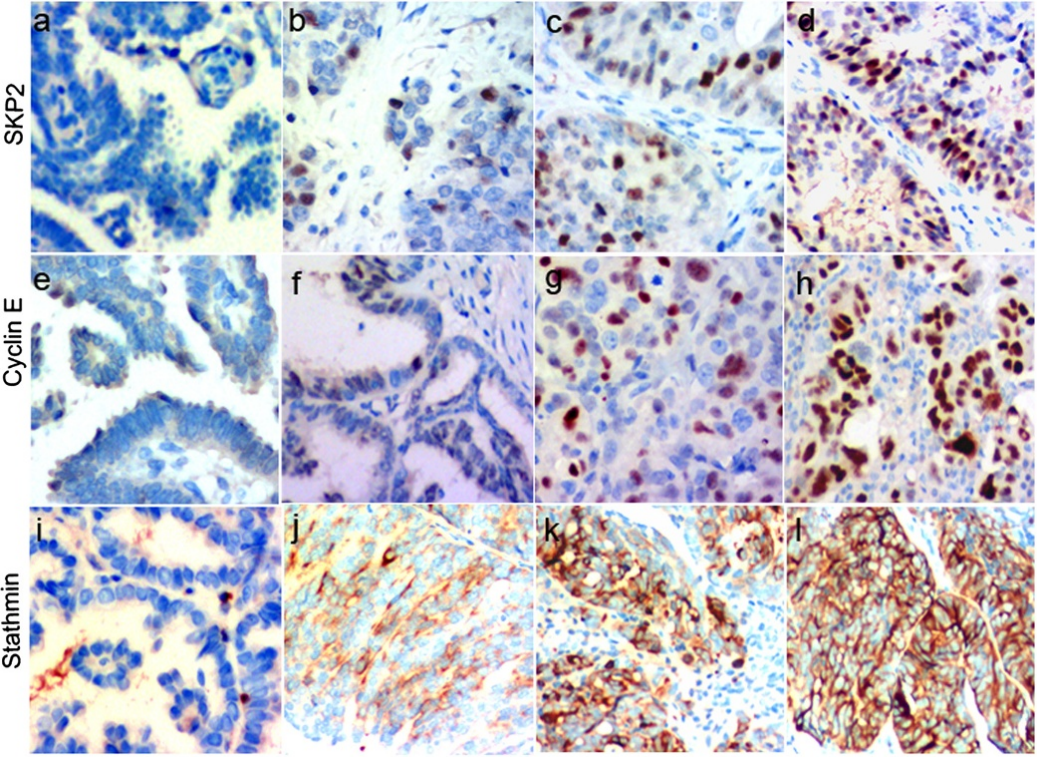
**The expression of SKP2, cyclin E, and stathmin in OSC.** SKP2 expression in **(a)** borderline ovarian serous cystadenoma, **(b)** low-grade OSC, **(c)** high-grade OSC, and **(d)** metastatic foci (×200). Cyclin E expression in **(e)** borderline ovarian serous cystadenoma, **(f)** low-grade OSC, **(g)** high-grade OSC, and **(h)** metastatic foci (×200). Stathmin expression in **(i)** borderline ovarian serous cystadenoma, **(j)** low-grade OSC, **(k)** high-grade OSC, and **(l)** metastatic foci (×200).

Eighty formalin-fixed, paraffin-embedded ovarian serous tumor tissues including cystoadenoma, low-grade OSC and high-grade OSC and their metastatic foci were used to construct a tissue microarray. IHC staining of cyclin E, SKP2, and stathmin was performed on the microarray slides. As shown in Figure 3, positive SKP2 (Figure 3a–d) and cyclin E (Figure 3a–d) staining was present in the nucleus of tumor cells, whereas positive stathmin staining was detected in the cytoplasm (Figure 3i–l).

SKP2 (*χ*^2^ = 12.384, *P* = 0.006), cyclin E (*χ*^2^ = 17.985, *P* = 0.000), and stathmin (*χ*^2^ = 20.226, *P* = 0.000) staining indexes were significantly different among the 4 groups (Table 3). The metastatic cancer cells from high-grade OSC had the highest SKP2, cyclin E, and stathmin staining indexes and borderline serous cystadenoma had the lowest (Table 4). Statistical analysis showed that the expression of SKP2 (*Z* = -1.182, *P* = 0.237), cyclin E (*Z* = -2.670, *P* = 0.008), and stathmin (*Z* = -2.487, *P* = 0.013) was higher in metastatic tumors than in primary high-grade OSCs. The staining index for cyclin E and stathmin was significantly different between group I and group II (Table 4). The expression of SKP2 (*Z* = -2.450, *P* = 0.014), cyclin E (*Z* = -2.068, *P* = 0.039), and stathmin (*Z* = -0.295, *P* = 0.768) was higher in primary low-grade ovarian carcinoma without metastasis than in borderline serous cystadenoma. The differences in SKP2 and cyclin E expression were statistically significant (Table 5).

**Table 3:** **The differences of stathmin, cyclin E and SKP-2 expression in the four groups of human ovarian tumors**

	Group	n	SKP-2	Cyclin E	Stathmin
Primary ovarian tumor with metastasis	I	21	1.33 ± 1.55	2.57 ± 2.13	0.86 ± 1.93
Corresponding metastatic tumor	II	21	1.95 ± 1.74	4.42 ± 1.98	1.95 ± 2.15
Primary ovarian tumor without metastasis	III	26	0.88 ± 0.99	2.38 ± 0.46	0.27 ± 0.87
Borderline serous cystadenoma	IV	12	0.17 ± 0.38	1.00 ± 1.27	0.17 ± 0.57
*χ* ^2^			12.384	17.985	20.226
*P*			0.006	0.000	0.000

**Table 4:** **The differences of stathmin, cyclin E and SKP-2 expression in primary ovarian tumor and their corresponding metastatic tumor**

	Group	n	SKP-2	Cyclin E	Stathmin
Primary ovarian tumor with metastasis	I	21	1.33 ± 1.55	2.57 ± 2.13	0.86 ± 1.93
Corresponding metastatic tumor	II	21	1.95 ± 1.74	4.42 ± 1.98	1.95 ± 2.15
Z			-1.182	-2.670	-2.487
*P*			0.237	0.008	0.013

**Table 5:** **The differences of stathmin, cyclin E and SKP-2 expression in primary ovarian tumor without metastasis and borderline serous cystadenoma**

	Group	n	SKP-2	Cyclin E	Stathmin
Primary ovarian tumor without metastasis	III	26	0.88 ± 0.99	2.38 ± 0.46	0.27 ± 0.87
Borderline serous cystadenoma	IV	12	0.17 ± 0.38	1.00 ± 1.27	0.17 ± 0.57
*Z*			-2.450	-2.068	-0.295
*P*			0.014	0.039	0.768

### Correlation among SKP2, cyclin E, and stathmin protein expression in OSC

To determine the association among SKP2, cyclin E, and stathmin protein expression in OSC, we performed a correlation analysis. Statistical analysis showed that the expression of SKP2 was positively correlated with cyclin E and stathmin expression. The correlation coefficient of SKP2 and cyclin E was 0.483, which was statistically significant (*P* = 0.001). SKP2 expression was also positively and significantly correlated with stathmin expression (correlation coefficient, 0.320; *P =* 0.028).

## Discussion

PGCCs contribute to solid tumor heterogeneity and play an important role in tumor initiation, metastasis and chemoresistance [10]. PGCCs are generally considered to be senescent or at the stage of mitotic catastrophe, our data demonstrated that these large cancer cells were actually live and generate the progeny cancer cells through budding [10, 24]. The PGCCs could form through endoreduplication or cell fusion, reverting to regular cancer cells through splitting, budding, or burst-like mechanisms commonly used by simple organisms. PGCCs divided asymmetrically and cycled slowly with a dynamic population [9, 10, 22]. They were positive for normal and cancer stem cell markers, and differentiated into adipose, cartilage, and bone. PGCCs induced by CoCl_2_ exhibit cancer stem cell properties and generate daughter cells via asymmetric division [10]. Daughter cells of PGCCs possess mesenchymal phenotypes and show stronger migratory and invasive ability than untreated diploid cells. The expression of cell cycle regulatory proteins including Cyclin E, SKP2, Stathmin, phosphorylated AKT, protein kinase C, phosphoglycerate kinase 1, p38, and mitogen-activated protein kinase in PGCCs with budding daughter cells are higher than those in untreated diploid cells. Recent studies have made great progress in dissecting the role of cell cycle regulatory mechanisms in carcinogenesis and tumors metastasis. Impaired cell cycle regulation is thought to be actively involved in all stages of carcinogenesis. Cell cycle proteins (cyclins), CDKs, and CDKIs are the main cell cycle regulators during tumor progression [25]. In the present study, we investigated the expression of three cell cycle-related factors including cyclin E, SKP2, and stathmin, in OSC and their association with the OSC grade.

Cyclin E, an important member of the cyclin family, interacts with CDK2 to form a functional complex that promotes cell cycle progression. Cyclin E overexpression has been detected in various cancers, including breast cancer [26], gastric cancer [27], and colorectal cancer [28]. Session, et al. found that the expression of cyclin E was significantly higher in OC tissues than in benign ovarian tumors [29]. Furthermore, cyclin E expression was significantly upregulated in metastatic lymph nodes and ascites. Together, these findings indicate that overexpression of cyclin E is positively associated with OC development and invasion. Our study showed that cyclin E is upregulated in high-grade OSCs compared with low-grade OSCs and borderline ovarian serous cystadenomas. We also found that cyclin E expression was significantly higher in metastatic foci than in primary high-grade OSCs.

Increasing biochemical and genetic evidence suggests that SKP2 is involved in multiple stages of the cell cycle [30–32]. SKP2 specifically recognizes phosphorylated substrates and induces ubiquitin-mediated degradation [33, 34]. Gstaiger showed that cotransfection of SKP2 and H-Ras significantly increased tumor formation in an animal model [35]. Studies have shown that SKP2 overexpression was positively correlated with the histological grade of malignant carcinomas. Fotovati et al. reported that SKP2 overexpression was positively associated with tumor progression and negatively associated with patient prognosis [36]. In the present study, we detected SKP2 protein expression in ovarian tumors. Furthermore, we demonstrated that SKP2 protein was upregulated in high-grade OSC and metastatic foci compared with low-grade OSCs and borderline serous cystadenoma. Our results suggest that SKP2 overexpression is associated with OSC metastasis and grade.

Stathmin promotes microtubule depolymerization or prevents microtubule polymerization in a phosphorylation-dependent manner. Stathmin is negatively regulated by phosphorylation. Accordingly, a less phosphorylable stathmin point mutant impaired extracellular matrix-induced microtubule stabilization and conferred a higher invasive potential [37]. Belletti et al. reported that overexpression of stathmin protein promoted sarcoma cell migration into adjacent local tissues and metastasis to distant organs [37]. Singer et al. reported that overexpression of stathmin accelerated the proliferation of non-small cell lung cancer cells and promoted their invasion and migration into the stroma [38]. Wei et al. showed that the expression of stathmin was high in OC cells, particularly in metastatic tumor cells [16]. Our results showed that the metastatic foci of high-grade OSCs had the highest expression of stathmin, which was positively correlated with SKP2 expression.

Few studies have investigated the relationship between the formation of PGCCs and the expression of cell cycle-related proteins cyclin E, SKP2, and stathmin in OSC. Cyclin E is among the main limiting factors controlling S phase entry of cells in G1 phase [39]. SKP2 helps cyclin E passing G1 checkpoint. Overexpressed SKP2 could combine with P27 to stimulate P27 ubiquitination and degradation via the ubiquitin-proteasome pathway [40]. Nelsen reported that co-transfection of cyclin E and SKP2 promoted S phase entry, DNA replication, and proliferation of liver cells [41]. The results of our study showed that the expression of cyclin E was positively correlated with the expression of SKP2 in OSC tissues. The expression of cyclin E reaches a peak in the late G1 or S phase and is absent in the G2/M phase. This indicates that cyclin E is not involved in the regulation of the G2/M phase, whereas SKP2 and stathmin play an important role in this phase. Stathmin phosphorylation/dephosphorylation controls cell cycle and cell motility. Stathmin is activated by simultaneous phosphorylation at the third or fourth phosphorylation sites in the G2/M phase. This step is essential for functional stathmin to facilitate cell transition from the G2 to M phase [42]. P27 interacts with stathmin to disrupt stathmin binding to tubulin, thereby inhibiting cell movement and microtubule polymerization. Upregulation of P27 in cancer cells inhibits stathmin protein expression to prevent the separation of stathmin from microtubules and promote the proliferative potential of cancer cells. SKP2 degrades P27 protein through ubiquitination, which promotes the expression of stathmin protein by reducing P27 inhibition [43, 44].

## Conclusions

The current study serves as the rationale for further investigation of the role of cyclin E, SKP2, and stathmin protein in the development and metastasis of OC. Our study suggests that these cell cycle-related proteins may represent useful prognostic and metastatic indicators for OC patients.

## Electronic supplementary material

Additional file 1: Table S1: Conventional TNM staging system of the ovarian carcinomas. (DOC 40 KB)
