# Supplementary material for: Polyploid giant cancer cells with budding and the expression of cyclin E, S-phase kinase-associated protein 2, stathmin associated with the grading and metastasis in serous ovarian tumor
Source: BMC Cancer. 2014 Aug 8;14:576. doi: 10.1186/1471-2407-14-576 (PMC4137091; doi:10.1186/1471-2407-14-576)
Supplement: Supplementary file 1 — Additional file 1: Table S1: Conventional TNM staging system of the ovarian carcinomas. (DOC 40 KB) [file 12885_2014_4766_MOESM1_ESM.doc]

**Additional files**

**Additional file 1 –Supplementary table 1. Conventional TNM staging system of the ovarian carcinomas.**

| Primary ovarian tumor with metastasis | | Primary ovarian tumor without metastasis | |
| --- | --- | --- | --- |
| No. |  | No. |  |
| 1 | T3cN1M0 | 1 | T1cN0M0 |
| 2 | T3cN1M0 | 2 | T1aN0M0 |
| 3 | T3cN1M0 | 3 | T1aN0M0 |
| 4 | T3cN1M1 | 4 | T1aN0M0 |
| 5 | T1bN1M0 | 5 | T2aN0M0 |
| 6 | T3cN1M0 | 6 | T1cN0M0 |
| 7 | T3cN1M0 | 7 | T1cN0M0 |
| 8 | T3cN1M0 | 8 | T1aN0M0 |
| 9 | T3bN1M0 | 9 | T1cN0M0 |
| 10 | T2bN1M0 | 10 | T1aN0M0 |
| 11 | T3bN1M1 | 11 | T1aN0M0 |
| 12 | T1cN1M0 | 12 | T1aN0M0 |
| 13 | T2bN1M0 | 13 | T1cN0M0 |
| 14 | T2bN1M0 | 14 | T1bN0M0 |
| 15 | T3cN1M1 | 15 | T3cN0M0 |
| 16 | T2bN1M0 | 16 | T1aN0M0 |
| 17 | T3aN1M0 | 17 | T1bN0M0 |
| 18 | T3aN1M0 | 18 | T3aN0M0 |
| 19 | T1bN1M0 | 19 | T1bN0M0 |
| 20 | T3cN1M0 | 20 | T1aN0M0 |
| 21 | T3cN1M0 | 21 | T1aN0M0 |
|  |  | 22 | T1bN0M0 |
|  |  | 23 | T1bN0M0 |
|  |  | 24 | T1aN0M0 |
|  |  | 25 | T1aN0M0 |
|  |  | 26 | T1aN0M0 |
